# Supplementary material for: Rapid Detection of Escherichia coli Antibiotic Susceptibility Using Live/Dead Spectrometry for Lytic Agents
Source: Microorganisms. 2021 Apr 26;9(5):924. doi: 10.3390/microorganisms9050924 (PMC8147107; doi:10.3390/microorganisms9050924)
Supplement: Supplementary file 1 [file microorganisms-09-00924-s001.zip › microorganisms-1163562-supplementary.pdf]

Supplementary Material

1 Supplementary Data

Supplementary Material should be uploaded separately on submission. Please include any supplementary data, figures and/or tables. All supplementary files are deposited to FigShare for permanent storage and receive a DOI.

Supplementary material is not typeset so please ensure that all information is clearly presented, the appropriate caption is included in the file and not in the manuscript, and that the style conforms to the rest of the article. To avoid discrepancies between the published article and the supplementary material, please do not add the title, author list, affiliations or correspondence in the supplementary files.

2 Supplementary Figures and Tables

Supplementary Table S1. Experimentally determined MIC and MBC of antibiotics against *E. coli*.

	Ampicillin		Polymyxin B		Ciprofloxacin		Chloramphenicol	
	MIC	MBC	MIC	MBC	MIC	MBC	MIC	MBC
Repeat 1	32	64	4	4	0.25	0.25	16	-
Repeat 2	32	64	4	4	0.25	0.25	16	-
Repeat 3	64	64	4	4	0.25	0.25	8	-
Repeat 4	32	64	4	4	0.25	0.25	16	-
**Median**	**32**	**64**	**4**	**4**	**0.25**	**0.25**	**16**	**N/A**

To determine the MIC (μg/ml), ~ 1×10^8 CFU/ml of *E. coli* was challenged with a doubling dilution of ampicillin, polymyxin B, ciprofloxacin, and chloramphenicol in a 96-well plate and incubated for 20 h at 37 ℃. The lowest concentration of antibiotic that prevented growth was determined to be the MIC. For MBC (μg/ml) determination, 10 μl of culture from the wells that did not show growth was

spread in triplicate onto TSA plates, which were incubated for 16 h at 37 ℃. The lowest concentration of antibiotic that resulted in no growth on the plates was determined to be the MBC.

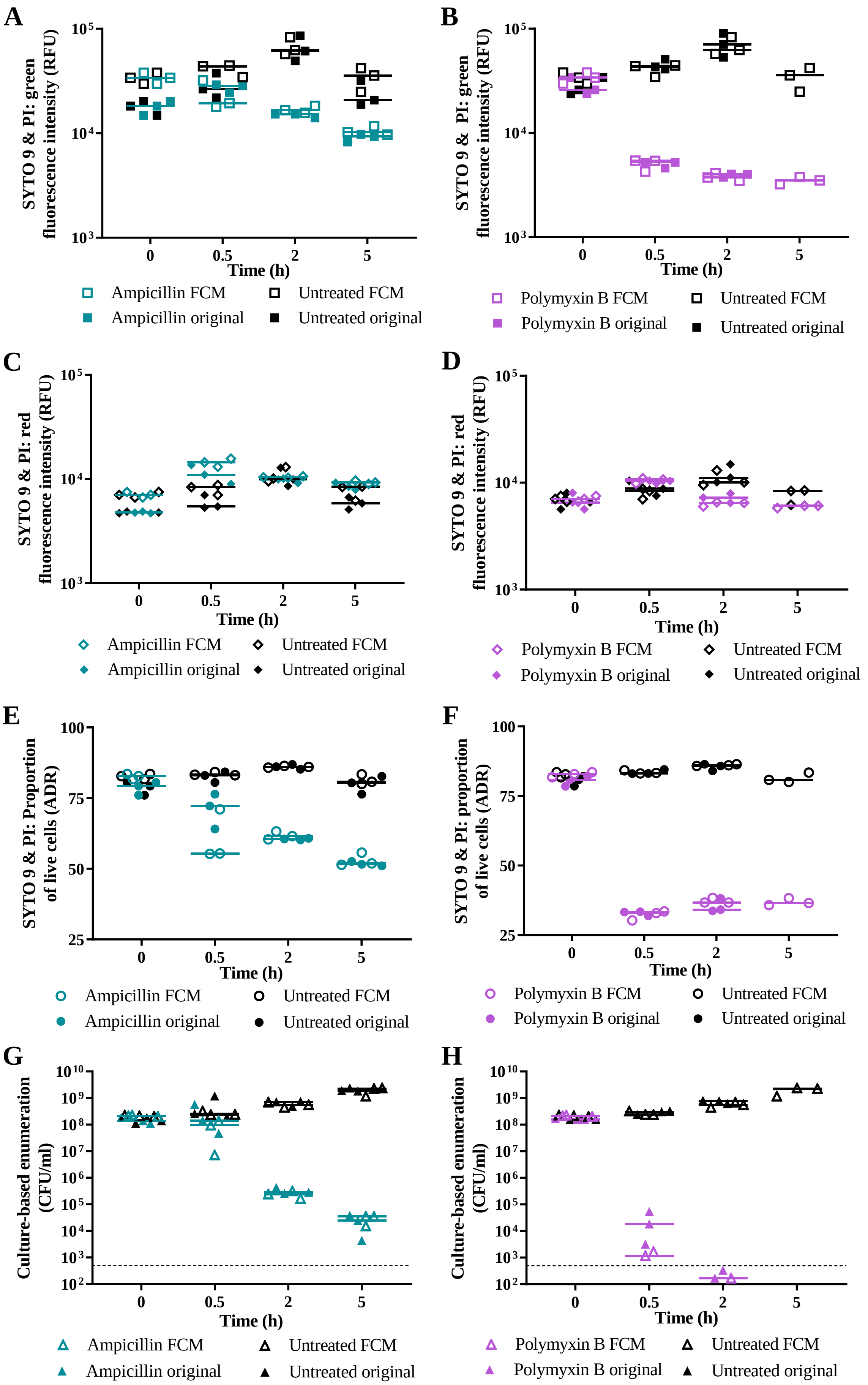
A
SYTO 9 & PI: green fluorescence intensity (RFU)
Time (h)
Ampicillin FCM
Ampicillin original
Untreated FCM
Untreated original
B
SYTO 9 & PI: green fluorescence intensity (RFU)
Time (h)
Polymyxin B FCM
Polymyxin B original
Untreated FCM
Untreated original
C
SYTO 9 & PI: red fluorescence intensity (RFU)
Time (h)
Ampicillin FCM
Ampicillin original
Untreated FCM
Untreated original
D
SYTO 9 & PI: red fluorescence intensity (RFU)
Time (h)
Polymyxin B FCM
Polymyxin B original
Untreated FCM
Untreated original
E
SYTO 9 & PI: Proportion of live cells (ADR)
Time (h)
Ampicillin FCM
Ampicillin original
Untreated FCM
Untreated original
F
SYTO 9 & PI: proportion of live cells (ADR)
Time (h)
Polymyxin B FCM
Polymyxin B original
Untreated FCM
Untreated original
G
Culture-based enumeration (CFU/ml)
Time (h)
Ampicillin FCM
Ampicillin original
Untreated FCM
Untreated original
H
Culture-based enumeration (CFU/ml)
Time (h)
Polymyxin B FCM
Polymyxin B original
Untreated FCM
Untreated original

Supplementary Figure S1. A comparison between live/dead spectrometry and viable cell plate counts of *E. coli* treated with lytic antibiotics from the original experiments and the flow cytometry experiments. At 0 h, 0.5 h, 2 h, and 5 h time points, viability of *E. coli* MG1655 treated with ~ 1 × MBC of ampicillin (teal), polymyxin B (purple), and an untreated control (black) was determined by measuring fluorescence SYTO 9- & PI-stained cultures using the Optrode (A – F) and by culture-based enumeration (G, H). Fluorescence intensities were obtained from integrating 505 – 515 nm for green emissions and 600 – 610 nm for red emissions. The proportion of live cells in the sample population was determined by the impact of the quenching/enhancement dye interaction on green emissions (A, B), level of red emissions (C, D), and the adjusted dye ratio (ADR; E, F). The closed data points are from the original experiments while the open data points are from the flow cytometry (FCM) experiments. The limit of detection culture-based enumeration is 500 CFU/ml (dashed line). Data presented is from three biological replicates with a line plotted at the median. No statistically significance differences were found between the original experiments and the FCM experiments (two-way RM ANOVA, P value: less than 0.05, Tukey multiple comparisons test).

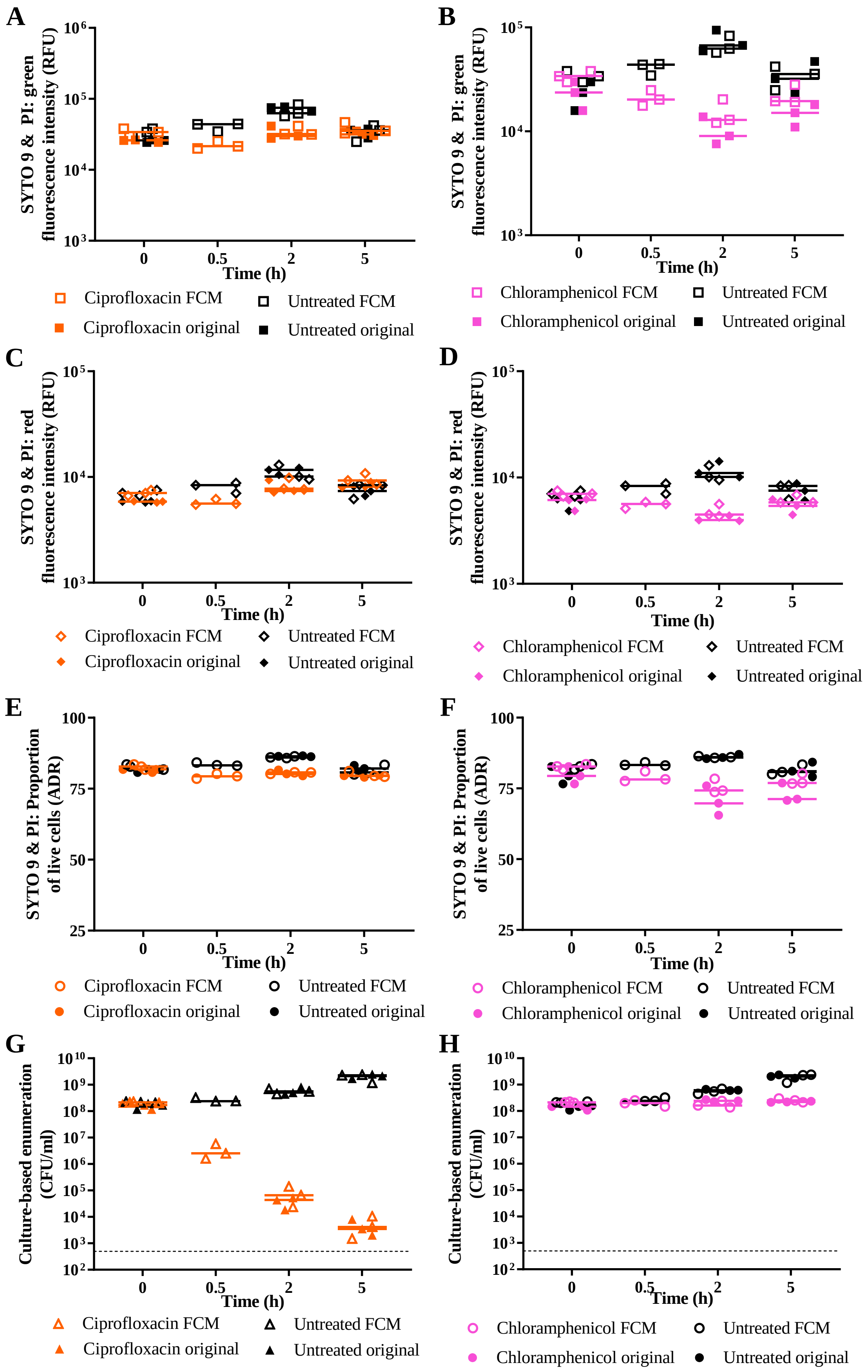
A
B
C
D
E
F
G
H
SYTO 9 & PI: green fluorescence intensity (RFU)
SYTO 9 & PI: red fluorescence intensity (RFU)
SYTO 9 & PI: Proportion of live cells (ADR)
Culture-based enumeration (CFU/ml)
Time (h)
0
0.5
2
5
Ciprofloxacin FCM
Ciprofloxacin original
Chloramphenicol FCM
Chloramphenicol original
Untreated FCM
Untreated original

Supplementary Figure S2. A comparison between live/dead spectrometry and viable cell plate counts of *E. coli* treated with non-lytic antibiotics from the original experiments and the flow cytometry experiments. At 0 h, 0.5 h, 2 h, and 5 h time points, viability of *E. coli* MG1655 treated with ~ 1 × MBC of ciprofloxacin (orange), chloramphenicol (pink), and an untreated control (black) was determined by measuring fluorescence SYTO 9- & PI-stained cultures using the Optrode (A – F) and by culture-based enumeration (G, H). Fluorescence intensities were obtained from integrating 505 – 515 nm for green emissions and 600 – 610 nm for red emissions. The proportion of live cells in the sample population was determined by the impact of the quenching/enhancement dye interaction on green emissions (A, B), level of red emissions (C, D), and the adjusted dye ratio (ADR; E, F). The closed data points are from the original experiments while the open data points are from the flow cytometry (FCM) experiments. The limit of detection culture-based enumeration is 500 CFU/ml (dashed line). Data presented is from three biological replicates with a line plotted at the median. No statistically significance differences were found between the original experiments and the FCM experiments (two-way RM ANOVA, P value: less than 0.05, Tukey multiple comparisons test).
